# Removing politics from innovations that improve food security

**DOI:** 10.1007/s11248-021-00261-y

**Published:** 2021-05-30

**Authors:** Stuart J. Smyth, Alan McHughen, Jon Entine, Drew Kershen, Carl Ramage, Wayne Parrott

**Affiliations:** 1 Department of Agricultural and Resource Economics, University of Saskatchewan, Saskatoon, SK Canada; 2 Department of Botany and Plant Sciences, University of California Riverside, Riverside, CA USA; 3 Genetic Literacy Project, Cincinnati, OH USA; 4 College of Law, University of Oklahoma, Norman, OK USA; 5 Office of the Deputy Vice-Chancellor, La Trobe University, Melbourne, VIC Australia; 6 Department of Crop and Soil Sciences, Institute of Plant Breeding, Genetics & Genomics, University of Georgia, Athens, GA USA

**Keywords:** Biosafety, Genome editing, Genetic modification, Regulation, Risk, Science-based

## Abstract

Genetically modified (GM) organisms and crops have been a feature of food production for over 30 years. Despite extensive science-based risk assessment, the public and many politicians remain concerned with the genetic manipulation of crops, particularly food crops. Many governments have addressed public concern through biosafety legislation and regulatory frameworks that identify and regulate risks to ensure human health and environmental safety. These domestic regulatory frameworks align to international scientific risk assessment methodologies on a case-by-case basis. Regulatory agencies in 70 countries around the world have conducted in excess of 4400 risk assessments, all reaching the same conclusion: GM crops and foods that have been assessed provide no greater risk to human health or the environment than non-GM crops and foods. Yet, while the science regarding the safety of GM crops and food appears conclusive and societal benefits have been globally demonstrated, the use of innovative products have only contributed minimal improvements to global food security. Regrettably, politically-motivated regulatory barriers are currently being implemented with the next genomic innovation, genome editing, the implications of which are also discussed in this article. A decade of reduced global food insecurity was witnessed from 2005 to 2015, but regrettably, the figure has subsequently risen. Why is this the case? Reasons have been attributed to climate variability, biotic and abiotic stresses, lack of access to innovative technologies and political interference in decision making processes. This commentary highlights how political interference in the regulatory approval process of GM crops is adversely affecting the adoption of innovative, yield enhancing crop varieties, thereby limiting food security opportunities in food insecure economies.

## Introduction

The United Nations Millennium Declaration, signed in September 2000, committed world leaders to combat poverty, hunger, disease, illiteracy, environmental degradation and discrimination against women. The Millennium Development Goals derived from this Declaration saw concerted effort and progress in reducing the number of food insecure from 825 to 629 million (FAO 2020). Unfortunately, global food insecurity is once again on the rise with 2019 data indicating the number had risen to 688 million and is projected to increase until at least 2030, when it is estimated the number of food insecure will reach 841 million. If these projections are realized, the world will be in a poorer position in terms of food security in 2030, than it was twenty-five years previously. In addition to a reduction in food security, the Food and Agriculture Organization (FAO) estimates that a further 2 billion people lack regular access to sufficiently nutritious food. The combination of people that are food insecure and nutrient deficient now represents one-third of the global population.

It is possible the numbers reported could be even higher, given the recent crop production challenges experienced in Africa due to locust and fall army worm incursions, as well as the adverse effects from changes in climactic conditions, all of which have been further impacted by the challenges of the Covid-19 pandemic. Bebber et al. (2014) suggest that many agricultural production countries could be saturated with plant pests and pathogens by mid-century, therefore it is quite possible that the decade of the 2020s could witness a dramatic global decline in food security.

While nature continues its relentless attacks on food production across the world, technologies that have safely been used for over 25 years, capable of contributing to increasing food production and security, continue to be denied approval and acceptance. In 2019, genetically modified (GM) crops were produced on 190 million hectares in 29 countries, with a further 42 importing GM crops for livestock feed and human food purposes (ISAAA 2020). Yield benefits of GM crops are significantly higher than non-GM crops, an average of 22% through the first 15 years of GM crop production (Klümper and Qaim 2014). The yield advantage of GM crops over organic crops is even more pronounced as organic crops experience a 30% yield lag compared to conventional, non-GM crops (Savage 2015). With organic yields producing only 70% of what a conventional crop can yield and with GM crops producing 22% above conventional yields, GM crops out-yield organic crops by 70%.

Approved GM crops have undergone 4485 risk assessments in the various producing and importing countries (IAAAS 2020). The results of the risk assessments have confirmed that the potential harm from the production and human consumption of an approved GM crop do not differ from the risks of producing the non-GM counterpart. Further, a review of data involving livestock GM crop feeding trial data involving over 100 billion head of livestock, found no difference in nutritional profile or health (van Eenennamm and Young 2014). Science-based risk assessments have quantified the safety of GM crops for human consumption and economic impact assessments have quantified the yield increases. These assessments raise the question: why are safe, higher yielding crops not being more widely adopted and produced? The answer is stated simply: politics.

Political interference by environmental non-governmental organizations (ENGOs) through campaigns of misinformation (Twyman et al. 2009; GLP 2020; CAST 2020; Ryan et al. 2020) have prevented the commercialization of GM crops in many food insecure countries (Berman et al. 2013). The cost of this political interference, in terms of adverse health effects and lives lost is staggering. Pelligrino et al. (2018) report the expression of cancer-causing mycotoxins are 30% lower in GM corn. Each year of delay in the approval of GM cowpea in Nigeria was estimated to result in the needless loss of up to 3000 lives (Wesseler et al. 2017). Delays in the commercialization of Golden Rice in India are estimated to have resulted in the loss of 1.4 million lives between 2004 and 2014 (Wesseler and Zilberman 2014).

Political interference has harmed human food security and health over the 25-year period GM crops have been available, but now also threatens the many opportunities offered by genome editing technologies (e.g., biofortification, plant disease, insect resistance, drought tolerance, increased photosynthesis, improved nitrogen use efficiency). Additionally, as climate changes adversely affect yields, new crop varieties capable of maintaining high yields are unable to be developed or commercialized. This commentary highlights the challenges and costs that political interference has created, not only in the regulatory systems of countries, but in the agriculture industry’s contributions to reducing the levels and rates of food insecurity. Genome editing techniques are being applied to a wide variety of food crops, with experimental yield increase results showing significant potential for genome-edited (GEd) crops to contribute to reducing food insecurity, as well as combating the effects of changing climates.

### Contributions of biotechnology plant breeding to improved food security

Abiotic and biotic stresses on crops reduce yields. Numerous studies in Africa have shown the devastating effects uncontrolled and less-than-optimally-controlled weeds can have on crop yield (Akobundu 1980; Carson 1976; Chikoye et al. 2004; Atera 2012). According to Kent et al. (2001), the principal limiting factor to farm size in Africa is the number of necessary weedings during the period following planting and Marais (1992) argues that poor weed control is the single biggest contributor to low maize yields for smallholder farmers in Africa. In 2020, plagues of locusts in eastern Africa devastated crops destined for human food purposes, with one estimate indicating that one swarm of locusts can eat as much in one day as 35,000 people (Gilliland 2020). Without control mechanisms, such as herbicides and insecticides, millions can be at risk of food impoverishment. The advantage of herbicide tolerant and insect resistant GM crops is that farmers no longer have to control weeds by hand and insecticide applications are significantly reduced.

The adoption of GM corn in South Africa was found to reduce the amount of time that women spent hand weeding fields, defined as drudgery, which contributed to improved food security. Gouse et al. (2016) surveyed female landholders over a three-year period, finding they spent 10–12 fewer days hand weeding, per season. Part of this time saving was reallocated to larger vegetable gardens as more time was available to haul water, increasing the household’s food security. In 2020, Nigeria approved the commercial production of pod borer resistant cowpea, announcing 10 tonnes of seed would be available, enough seed for 10,000 farmers (National Accord Newspaper 2021). Previous research indicates that untreated cowpea production yielded 76 kg/ha, yet through the use of insecticides, yields rose to 1382 kg/ha (Raheja 1986). With the commercialization of GM Bt cowpea, insecticide cost reductions would be expected based on commercialization results in other Bt crops. Further reductions in insect damage would contribute to additional yield increases. One estimate indicates that the yield increase from Bt cowpea could be as high as 25% (Bett et al. 2017).

GM crops have been shown to have yield increasing benefits due to the reduction in abiotic and biotic stresses. Shelton et al. (2020) examined the adoption of Bt brinjal in Bangladesh, finding yield increases of 20%, compared to non-Bt brinjal. A similar assessment of GM corn adoption in Vietnam, found yield increases of 30% (Brookes and Dinh 2021). With higher yields from GM crops being confirmed, there is also evidence they contribute to reductions in poverty, which is the number one objective of the United Nation’s Sustainable Development Goals.^1^ In an assessment of the household income impacts of GM Bt cotton adoption and production in India, Subramanian and Qaim (2010) found that the poorest households in the survey, those living on less than US$2 per day, experienced an increase of 134%. The adoption of GM corn in the Philippines has observed similar benefits, with household incomes increasing by 50%, rising from US$400 per year to US$600 per year (Yorobe and Smale 2012).

GM Bt cotton adoption in Colombia has provided economic and environmental benefits between 2003 and 2015 that include: reduced water consumption by 119 million liters (enough for 2.7 million people); reduced diesel use by 1.8 million liters; 4.7 million fewer tonnes of carbon emissions (equivalent to preserving 34,500 trees); and 66 fewer tonnes of chemicals applied (Céleres 2015). The 2008 adoption of GM corn in Colombia has been estimated to have reduced water use by 240 L per hectare annually. Water conservation resulting from GM crops, can be reallocated to other food crop productions, contributing to improved food security. A farm survey on GM corn production in Honduras, quantified yield increases of 50% (Macall et al. 2020). An assessment of GM white corn field trials in El Salvador, found a yield increase of 18% (Macall and Smyth 2020). The adoption of GM soybeans in South America, has resulted in many farmers being able to produce two crops per year, whereas only one was previously possible (Brookes and Barfoot 2018). These farmers are able to plant and harvest a crop of soybeans, which is then immediately followed by the planting and harvesting of a wheat crop, raising farm incomes and contributing to increased food security.

While the advantages and benefits of commercializing and producing GM crops are increasingly being quantified, the lack of sound alternatives or benchmarks to discern what the impacts of not adopting GM crops are, is lacking. One study that examined the economic and environmental costs of not adopting GM crops, found the costs to be significant. Biden et al. (2018) compared GM canola adoption in Western Canada with Australia, where GM canola had been approved in 2004, but experienced moratoriums in the various canola producing states that lasted for periods of several years. In examining the period from 2004 to 2014, the authors found the failure to adopt GM canola in Australia as soon as it was available, resulted in the application of an additional 6.5 million kg of chemicals; 7 million additional field passes were made, requiring 8.7 million liters of diesel; 24 million kg of greenhouse gases were released through the additional field passes; the environmental impact of the additional chemicals applied was 14% higher; and Australian farmers lost the opportunity to increase their farm revenues by A$485 million.

GM crops have been proven to successfully combat the challenges caused by abiotic and biotic stresses, ensuring that the supply of food has at least been stable, if not increased. The majority of GM crops presently produced exhibit the trait for herbicide tolerance or insect resistance, or in increasing instances, both traits. As climates change over the coming years, additional innovations will be required to ensure the production of food continues to increase, as if yields remain constant, increases in the global population will result in greater numbers of food insecure. As plant breeding technologies transition from transgenics, where ‘foreign’ genes are inserted into varieties, to genome editing, with its targeted and controlled mutations within the genome, there is significant potential for GEd crop varieties to be more resilient in the face of climate changes.

Genome-edited crops are demonstrating evidence of their ability to contribute to improved food security (Qaim 2020). Preliminary yield results from field trials involving GEd varieties highlight the level of potential as rice yields increased by 20%, when edited for increased heat tolerance (Chen et al. 2020). Sorghum edited specifically for yield increases demonstrated a 200% increase in experimental trials (Gladman et al. 2019). Nutritional enhancements are an essential component of improved food security (Farre et al. 2011), with increases in vitamin E in sweet corn being demonstrated (Xiao et al. 2020), while sustainability can be improved through increased water-use efficiency (Glowacka et al. 2018). Ku and Ha (2020) undertook a review of genome editing applications to enhance nutritional aspects of food crops, indicating research is progressing involving the three staple crops of rice, wheat and corn, but additionally including potatoes, tomatoes and grapes. Lassoued et al. (2019) estimate that if GEd crops are required to be regulated as equivalent to GM crops, regulatory approval times will require an additional nine years, with a cost increase of US$14 million.

Improving food security in the coming decades, is a global priority as the FAO has estimated that yield increases of 70% are required, with increases of 100% required in some countries (FAO 2005). With GM crops having undergone thousands of risk assessments in over 70 countries, without the quantification of a single risk factor being any different from an equivalent non-GM crop variety, the time has come to set aside the politics from food security and begin the logical inclusion of these safe, higher yielding crop varieties as part of the global strategy to ensure the UN’s top two SDGs, poverty reduction and zero hunger, are achieved. As climate changes have been predicted to have an increased adverse impact on food production, new plant breeding technologies that are demonstrating preliminary yield increases, should not be subject to the politically motivated campaigns to restrict or ban these technologies. The future of reducing food security relies on having a well-stocked toolbox, capable of offering higher yielding varieties by every means possible.

### The science and politics of regulating future food security innovations

A number of scientific societies, academic institutions and regulatory agencies around the world have investigated various regulatory, safety and policy issues surrounding genome editing techniques, making science-based recommendations to policymakers formulating regulations (Academy of Science of South Africa 2017; CAST 2018; EASAC 2015; EFSA 2012, 2015; JRC 2011; Leopoldina 2015; USDA 2018; VIB 2018). The common conclusions in these opinions by scientific organizations include imposing regulatory scrutiny based on the documented risks of the product, rather than on the process used to breed them. Another common conclusion holds that many products of genome editing may warrant no additional regulation beyond those for conventional plants, if they could have been generated using ‘conventional’ methods of breeding or lack DNA segments over 20 base pairs originating in incompatible species.

Regrettably, the science-based recommendations to achieve reasonable safety outcomes are often ignored or marginalized in favor of political agendas (Masip et al. 2013; Smyth 2020). These are often based on the flawed assumption that genetic changes made in a laboratory are inherently riskier than the same changes made by nature, as genes naturally mutate from one generation to the next. Furthermore, genetic changes directed by humans in a lab are assumed, again falsely, to be inherently ‘unnatural’ which also implies higher risk. Indeed, some of the statutory and regulatory language is patently unscientific. One common example is the European Union (EU) definition of ‘GMO’ as being “an organism … in which the genetic material has been altered in a way that does not occur naturally by mating and/or natural recombination”, thus capturing all agricultural products of genetic modification, including those using Agrobacterium as a vector (EU Directive 2001/18/EC). However, Kyndt et al. (2015) has shown ordinary, natural sweet potatoes carry Agrobacterium DNA fragments in its genome, inserted by a natural recombination process thousands of years ago. Clearly, genome transfer, even across the ‘species barrier’ is a natural process.

The EU is arguably the most conflicted region in terms of allowing politics to govern the regulatory and approval process for GM crops. The establishment of the European Food Safety Authority in 2002 by the European Union to provide independent scientific evidence and advice about food safety and potential risks, decoupled the risk assessment process from the variety approval process. The result of this has been that the risk assessment process is science-based and has consistently delivered risk assessment approval decisions for GM crops, identical in conclusions to risk assessments in all other GM crop producing countries. However, the problem lies with the variety approval process, which resides with the European Commission’s Standing Committee on the Food Chain. It is this Committee that approves GM crop varieties for commercial planting and production in the EU. For any GM crop variety that has been scientifically found to have risks no greater than any of the conventional or induced mutation crop varieties approved for production in the EU, a qualified majority vote must result from this Committee. A qualified majority vote within EU requirements means that 15 of the 27 EU Member States, constituting 65% of the total EU population, must approve the decision (Sabalza et al. 2011). The fact that only one GM crop variety has been approved for production in the EU since 2003, confirms the political basis for Committee votes. In fact, the Committee deliberately ignores the independent scientific advice provided by the very agency established to better inform the EU decision making process. Over 300 million euros have been invested in research projects by the European Commission, with the objective of assessing potential risks from GM crops. The results of these studies confirm “the efforts of more than 130 research projects, covering a period of more than 25 years of research, and involving more than 500 independent research groups, is that biotechnology, and in particular GMOs, are not per se more risky than e.g. conventional plant breeding technologies” (European Commission 2010). The failure to adopt GM crops in the EU at the same time and rate as other countries, has resulted in EU agriculture needlessly releasing an estimated 33 million additional tonnes of carbon dioxide per year (Kovak et al. 2021).

Recognizing the dire challenge facing EU agriculture, the European Commission’s Group of Chief Science Advisors (2019: 6) recommended, “revising the existing GMO Directive to reflect current knowledge and scientific evidence, in particular on gene editing and established techniques of genetic modification. This should be done with reference to other legislation relevant to food safety and environmental protection.” This message was subsequently reinforced in 2020, with the Union Européenne des Académies d’Agriculture stating that “the European Union cannot do without genetic engineering and that European regulations must take into account these new scientific advances in human therapeutic fields, which are universally accepted, as well as in terms of animal or plant health in the context of sustainable agriculture” (UEAA 2020). Efforts are underway within the EU that examine how genome editing technologies might be more efficiently regulated, as the Council of the European Union requested the European Commission submit a report addressing this by April 2021 (European Commission 2019). Clearly, Europe’s scientific community are publicly acknowledging that EU Directive 2001/18 is a barrier to R&D investment and innovative plant breeding technologies.

On 29 April 2021, the Commission submitted its report, indicating that the lack of clarity regarding the regulation of genome editing technologies creates regulatory uncertainty (European Commission 2021). The report confirms that EFSA has assessed GEd crop varieties, concluding the hazards from these varieties do not differ from conventionally developed crop varieties. Additionally, the report articulates that if the EU’s regulatory framework for GMOs is applied to GEd crops, this could lead to trade limitations, as well as putting EU farmers at a competitive disadvantage to farmers in other countries where GEd crops are being commercialized. The report concludes that if the existing GMO regulatory framework is applied to GEd varieties, it will act as a barrier to innovation, thereby recommending that the regulatory framework be revised such that it provide greater clarity, shift from a process-based to product-based system and be more uniformly applied.

Europe has already experienced a significant agricultural R&D investment decline. In the mid-1990s, Europe accounted for one-third of global agricultural R&D investments. By 2013, this had fallen to less than 10% (Phillips McDougal 2013). Europe’s regulatory framework has driven billions of euros of investment to jurisdictions with functioning regulatory systems. European scientists are now witnessing an identical regulatory approach to genome editing as to genetic modification and are rightfully concerned about what this means for Europe’s agricultural future. In July 2018, the Court of Justice of the European Union (CJEU) ruled that regardless of the fact that in most applications, genome editing technologies do not result in foreign DNA being inserted into a new variety, all products developed by genome editing would be required to be regulated as equivalents to GMOs. The innovation investment costs have been substantial as BASF and Bayer immediately announced they were relocating their genome editing research outside the EU. Following this, numerous other companies announced similar plans (Reuters 2018; Sikkema 2018).

The problem is not limited to the EU. Many jurisdictions are struggling to utilize legislation that is no longer fit for purpose in addressing or assessing the potential risk/harm posed by genetic changes to crop plants. The existing systems are unable to easily be amended to reflect over 25 years of safe use of GM crops or to allow for appropriate risk tiering. Much of the blame for these rigid, inflexible regulatory systems is due to the Cartagena Protocol on Biosafety (Smyth 2017). One significant barrier to crop variety innovation is Article 26 of the CPB, which allows countries to implement socio-economic considerations (SECs) into their regulatory frameworks for GM crops. Ludlow et al. (2014) identified that many of the potential SECs that could be included, lack proven, reliable methodologies and the required data often does not exist. Article 26 and the CPB thus become barriers to innovation. The lack of methodologies and data—combined with regulators with no experience pertaining to SEC assessments—results in substantial commercialization delays as well as GM variety approvals being denied. As part of the process of ensuring that most developing countries ratified and adopted the CPB, the EU used it as leverage for developing countries wanting preferential trade treatment. The World Trade Organization allows countries to provide lower tariff rates to specific countries for specific products, that are not available to other countries exporting similar products. The EU required any developing country that wanted lower tariff rates to implement the CPB (European Commission 2016). The EU deliberately utilized politically motivated rational for the adoption of the CPB by developing countries, preventing the commercialization of yield enhancing GM crops, thus perpetuating food insecurity.

To further the global rejection of GM crops, ENGOs have waged deliberate campaigns of misinformation (Ryan et al 2020; CAST 2020). The Genetic Literacy Project (2020) has extensively tracked the flow of money, estimating that as much as US$850 million was spent by ENGOs between 2012 and 2016. With the opposition to GM crops beginning in the late 1990s, billions of dollars have been wasted lobbying against proven, safe, yield enhancing crop varieties, that would have been better invested in research on improving crop varieties. Given the total lack of verifiable scientific quantification of risks from GM crop production and consumption, this opposition is entirely politically motivated. One example of this opposition has been the relentless campaign by Greenpeace against Golden Rice. Golden Rice has been biofortified to provide increased vitamin A, the lack of which leads to blindness and childhood death. Research by the International Rice Research Institute, the Philippine Rice Research Institute and the Bangladesh Rice Research Institute, indicates that 30–50% of vitamin A requirements could be provided by Golden Rice (Swamy et al. 2021). To illustrate the lack of humanity resulting from Greenpeace’s actions, 157 Nobel Laureates signed a public letter calling on Greenpeace to end their opposition to Golden Rice.^2^

The combination of the EU’s politically-based variety approval system and intensive misinformation and disinformation ENGO campaigns, have significantly contributed to food insecure countries basing their rejection of GM crops on the EU’s policy framework or ENGO messaging. Several examples are worth mentioning as just a sample of the misinformation and disinformation. In the early 2000s Greenpeace actively campaigned in Africa that if people consumed GM corn, it would make them sterile—maliciously false. ENGOs have consistently spread the false claim that Bt cotton in India has led to increased farmer suicides (Smyth 2020). ENGOs have routinely falsely claimed that biofortified rice is an attempt by multinational seed companies to seize control of rice production when, in fact, the biofortified Golden Rice is a public interest initiative from academic and governmental breeding programs. GM crops are produced and imported into over 70 countries, all without a single incidence of adverse impact to human health. Over the past 20 years, food insecure countries have rejected crop varieties that have been developed by biotechnology, which have higher yields, with none of these rejections grounded in scientific evidence. All of these GM crop variety rejections have been based on political preference, with the real life cost of higher food insecurity, increased malnourishment and premature death.

### Impacts of political interference on the future of food security

Improved food security will require all of the plant breeding technologies that are presently available—and those to come as molecular biology knowledge advances. GM Bt corn and cotton have been shown to have spill-over benefits, whereby neighboring non-GM, organic and agroecology practicing farmers experience lower pest populations, reducing chemical inputs and increasing yields for non-adopting farmers as well as adopting farmers (Hutchison et al. 2010; Huang et al. 2010). There are farmers and consumers that prefer to produce and consume food products marketed as natural or organic and the integration of multiple production technologies is essential in ensuring both producers and consumers have choice. Those that fail to appreciate or understand the importance of the inter-connectivity of agricultural crop and food product and seek to ban GM crop technologies, will in turn, reduce the yields of alternative crop production methods. The end result will be significantly lower levels of food production.

The politics of rejecting safe GM crops, has perhaps never been more glaringly apparent than in the 2002–03 drought in the African nations of Zambia and Zimbabwe. The United States, through the UN’s World Food Program, donated over 40,000 tons of corn, which included GM varieties (Manda 2003). Campaigns of misinformation spread by ENGOs, resulted in the political leaders of these countries trusting this information. In the case of Zambia, even though an estimated 30% of the country’s population faced starvation, the country’s leaders banned the donated GM corn. Zimbabwe rejected 10,000 tons of corn, but eventually allowed subsequent corn food aid donations to enter the country, regardless of whether GM corn was present. Paarlberg (2014) provides an excellent account of the political interference exerted by ENGOs, particularly those based in Europe, that led to these countries rejecting safe GM corn in the facing of starving populations. ENGOs have been so opposed to any research involving GM crops that they initiated a campaign to destroy research field trials involving GM crop varieties. This occurred predominantly in Europe, but campaigns were also carried out in many food insecure countries. In 2012, Kenya banned all GM corn imports over misinformation spread by ENGOs about GM corn being linked to the development of cancer (USDA 2012).

Political interference is preventing GM crops that have been produced in countries for years or decades, from being commercially produced in other countries. GM Bt brinjal has been proven to increase yield and reduce insecticide use in Bangladesh (Shelton et al. 2020), but is banned by farmers in neighboring India. In 2010, India’s government imposed a 10-year moratorium on GM crop approval. However, Indian farmers have learned of the experiences of Bangladesh farmers with Bt brinjal and have begun to illegally grow this crop in India (Haq 2019). Desperate for access to crops that are able to safely increase yields and reduce insecticide applications, Indian farmers are risking fines and jail sentences, to have access to Bt brinjal, defying the Indian government moratorium.

Other countries have also implemented bans against GM crops. Late in 2020, Peru extended its current ten-year GM crop production moratorium for a further 15 years (Montaguth 2020). The irony of the moratorium is that while domestic GM corn production is banned, Peru imported over 4 million tonnes of corn, a staple in Peruvian diets, from Argentina, where GM corn adoption is nearing 100%. Additionally, Peru spent over US$140 million on soy imports in 2019, 80% of which came from the USA, where GM soy is at near full adoption. Rather than allow farmers in Peru to have access to GM corn and soy, which would increase yields, the Peruvian government spends additional money, that could be better allocated to education or health needs of its citizens, on importing GM corn and soy from other countries. Chile also prevents its farmers from having access to GM crops, yet is extensively involved in the multiplication of GM seed for sale in other markets (Sanchez 2020). Between 2009 and 2018, over 1000 GM crop events were multiplied in Chile, none of which were available for Chilian farmers to produce.

The hypocrisy of the EU is perhaps no more glaring than in its import of GM corn and soy from the Americas to be used a livestock feed. Over the past decade, 2011–2021, the EU imports of corn range from 9 to 25 million tonnes, with soy imports ranging from 12 to 15 million tonnes (Index Mundi 2021a, b). The EU’s lack of willingness to allow GM corn and soy to be produced, other than the small amounts of production occurring in Portugal and Spain, instead results in the EU relying on imports from biodiverse rich regions of the world, resulting in the EU being responsible for 16% of global tropical deforestation (Koval et al. 2021). EFSA has undertaken risk assessments on the GM corn and soy varieties being imported livestock feed use in the EU, determining there are no environmental or human food safety risks from these imports. With GM corn and soy being safe to be imported into the EU for use as livestock feed, this raises numerous questions as to why it would not be safe for the EU to produce GM corn and soy domestically? Not only do these EU GM crop imports reduce tropical forests, millions of tonnes of additional greenhouse gases are released through the transportation of these commodities from the exporting country to the EU.

Moratoriums, prohibitions and bans have a lengthy history of failure, whether it be alcohol or tobacco, none have worked. The same is evident for bans on GM crops, farmers are connected to informed networks that share the results of GM crop production, driving demand for access to these yield enhancing crops. Politically motivated bans to the technology harm adopting countries in numerous ways. First, the supply of food is lower than what might otherwise be the case. Second, more time and money is spent by farmers without access to GM crops, to control weeds and pests that are proven to reduce yields. Third, governments that ban the domestic production of GM crops, turn around and import GM crops from other countries. Fourth, the money spent purchasing foreign produced GM crops supports farmers in these other markets, as well as reducing the amount of funding available for alternative domestic funding needs. Removing the politics from food security would allow governments to approve GM crops for domestic production, providing benefits to their own farmers and reducing the import costs of foreign commodities and products.

## Conclusions

The safety of GM crop production and consumption is scientifically quantified through the publication of hundreds of peer reviewed journal articles and numerous assessments by domestic scientific bodies, many of which have been done in the EU, all of which support the thousands of risk assessments conducted by independent federal regulatory agencies on GM crops that found no difference in risk between GM and non-GM varieties. With 25 years of GM crop consumption, all without any evidence of harm to human health or the environment, it is blatantly evident that opposition to the commercialization of GM crops is not grounded in scientific evidence, but rather political opportunism. If food insecure countries have any hope of overcoming their domestic food supply challenges, they need to firmly reject the deliberate ENGO campaigns of disinformation and misinformation and approve yield enhancing GM crops. ENGOs that remain opposed to GM crops with the abundance of evidence and safety are entering dubious territory as to the ethics of their sustained opposition. Perhaps the time has come for the world to ask, ‘Is it ethical to be opposed to increased food security from GM crops’?

While this commentary addresses the detrimental impacts the EU is having on improving food security, it is important to acknowledge that no country has avoided political opportunism and political interferences that have impeded the adoption of improved crops for food security. This commentary is focused upon the domestic laws and regulations impeding adoption and does not address the agricultural trade impacts of these domestic laws and regulations.

This commentary was written during the COVID-19 pandemic, in which nations have eagerly sought the benefits of biotechnology for the development of vaccines and treatments to protect the lives of their citizens. Yet, for those who are food insecure—either through lack of calories or malnutrition—food is the needed medicine. Laws and regulations that impede the adoption of safe and nutritious crops from biotechnology is, literally, the denial of the medicine the food insecure most need. Therefore, it bears repeating: ‘Is it ethical to be opposed to increased food security from GM crops’?

## Footnotes

1 https://sdgs.un.org/goals.

2 https://geneticliteracyproject.org/2020/05/05/anti-gmo-advocacy-funding-tracker-vast-network-of-donors-and-ngos-seed-doubt-about-crop-biotechnology/.
